# Uncemented monoblock trabecular metal posterior stabilized high-flex total knee arthroplasty: similar pattern of migration to the cruciate-retaining design — a prospective radiostereometric analysis (RSA) and clinical evaluation of 40 patients (49 knees) 60 years or younger with 9 years’ follow-up

**DOI:** 10.1080/17453674.2019.1626097

**Published:** 2019-06-18

**Authors:** Radoslaw Wojtowicz, Anders Henricson, Kjell G Nilsson, Sead Crnalic

**Affiliations:** a Department of Surgical and Perioperative Sciences, Umeå University, Umeå, Sweden;; b Department of Orthopaedics, Falu Hospital, Falun, Sweden

## Abstract

Background and purpose — Uncemented monoblock cruciate retaining (CR) trabecular metal (TM) tibial components in total knee arthroplasty (TKA) work well in the long-term perspective in patients ≤ 60 years. Younger persons expect nearly normal knee flexion after TKA, but CR implants generally achieve less knee flexion compared with posterior stabilized (PS) implants. Cemented PS implants have higher revision rate than CR implants. Can an uncemented monoblock PS TM implant be used safely in younger patients?

Patients and methods — 40 patients (49 knees) age ≤ 60 years with primary (20 knees) or posttraumatic osteoarthritis (OA) were operated with a high-flex TKA using an uncemented monoblock PS TM tibial component. Knees were evaluated with radiostereometric analysis (RSA) a mean 3 days (1–5) postoperatively, and thereafter at 6 weeks, 3 months, 1, 2, 5, and 9 years. Clinical outcome was measured with patient-related outcome measures (PROMs).

Results — The implants showed a pattern of migration with initial large migration followed by early stabilization lasting up to 9 years, a pattern known to be compatible with good long-term results. Clinical and radiological outcome was excellent with 38 of the 40 patients being satisfied or very satisfied with the procedure and bone apposition to the entire implant surface in 46 of 49 knees. Mean knee flexion was 130°. 1 knee was revised at 3 months due to medial tibial condyle collapse.

Interpretation — The uncemented monoblock PS TM implant works well in younger persons operated with TKA due to primary or secondary OA.

Normal knee flexion after TKA is often expected by patients, not least by younger patients. Posterior stabilized implants (PS) have consistently been shown to lead to greater flexion compared with cruciate retaining (CR) implants (Jacobs et al. 2005, Bercik et al. 2013, Li et al. 2014, Jiang et al. 2016). However, the revision rates for PS implants have been found to be higher compared with CR implants (Comfort et al. 2014, Vertullo et al. 2017), perhaps due to the higher constraint of the PS articulation leading to larger forces at the implant–bone interface (Catani et al. 2004).

Uncemented trabecular metal (TM) tibial implants have shown excellent clinical results in younger patients in the medium-term perspective (Kamath et al. 2011, Fernandez-Fairen et al. 2013). This may be due to inherent properties of the trabecular metal, seemingly enhancing biologic fixation (Rahbek et al. 2005, Bullens et al. 2010, Sagomonyants et al. 2011, Sambaziotis et al. 2012). In a radiostereometric (RSA) study of younger persons with 10-years’ follow-up, the CR uncemented monoblock TM implant displayed no loosening and a pattern of migration with early stabilization lasting up to 10 years, indicating good long-term performance as regards fixation (Henricson and Nilsson 2016). However, the mean postoperative knee flexion in that study was only 110°.

Posterior stabilized TM implants, thus, would be an attractive solution for the younger patient. However, there are few clinical long-term follow-up studies of such components in the literature, and to our knowledge no long-term RSA studies. Most RSA studies have focused on the magnitude of migration during the early postoperative years. Pijls et al. (2012b, 2018), in 2 systematic reviews of mostly cemented implants found that the magnitude of migration at 6 months and 1 year was associated with late revision. However, the pattern of migration over time is also important in determining long-term performance (Wilson et al. 2012, Henricson and Nilsson 2016, Molt et al. 2016, van Hamersveld et al. 2017, 2018).

We determined the migration, clinical, and radiological outcome of the NexGen LPS-Flex TM Monoblock (Zimmer, Warsaw, IN, USA) tibial component up to 9 years. The primary objective was to compare the pattern of migration of the PS implant with the CR variety of the same implant, results of which have been presented recently (Henricson and Nilsson 2016). Secondary objectives were to assess clinical results as measured by patient and clinician reported outcomes measures, postoperative knee motion, and radiological result.

## Patients and methods

**Figure 1.**
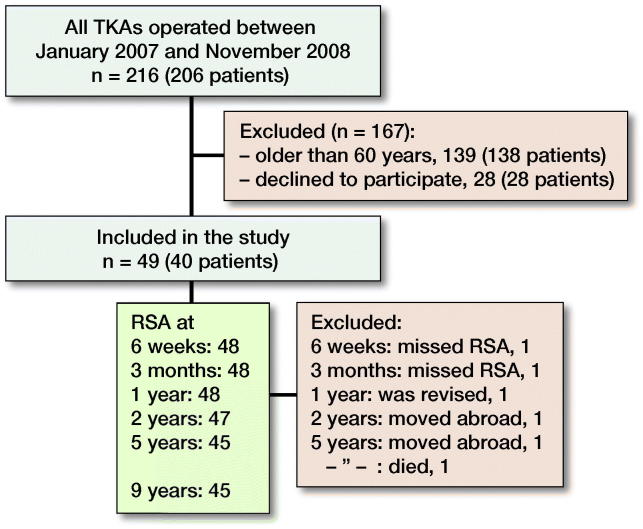
Flowchart of the patients. TKA = total knee arthroplasty, RSA = radiostereometric analysis.

The patients were recruited from the waiting list for knee arthroplasty at the Department of Orthopedics, Umeå University Hospital. Inclusion criteria were primary or secondary osteoarthritis (OA) with symptoms warranting knee arthroplasty, age 60 years or younger, and willingness and ability to participate in the study (Figure 1, Table 1). 29 knees were operated due to posttraumatic OA; 9 knees had had an anterior cruciate ligament lesion (4 operated, 5 non-operated), 10 meniscal surgery, 3 because of previous tibial osteotomy, 2 recurrent patellar luxation, 2 cartilage transplantation, and 1 tibial condyle fracture. 1 patient (2 knees) had OA secondary to psoriatic arthropathy.

**Table 1.** Preoperative clinical data

Factor	Mean	Median	Range
Age (years)	55	56	40–60
Weight (kg)	88	85	57–120
Length (cm)	171	169	153–198
BMI	30	30	21–43
Extension lag (°)	13	15	0–30
Flexion (°)	117	125	80–135
Range of motion (°)	104	110	60–135
Knee Society (KS) score **^a^**	17	14	0–48
KS function score **^a^**	48	50	25–55
Knee alignment (°) **^b^**	176	174	163–196

**^a^**Knee Society knee and Function score (max. 100).

**^b^**Hip Knee Ankle angle (varus < 180°, valgus > 180°).

The operations were performed under tourniquet by KGN. Ligaments were balanced as needed and the distal femoral and proximal tibial cuts were made using intramedullary guides. It was aimed to insert the tibial component at a right angle to the mechanical axis in the frontal plane and with 6–7° posterior tilt. All femoral components were cemented using Palacos with gentamicin bone cement (Heraeus Kulzer GmbH, Wehrheim, Germany). In 2 knees an all polyethylene patellar component was inserted. In 9 patients (6 women) both knees were operated, with a mean 6 months (5–16) between procedures. All patients received a PS Monoblock TM tibial component and a NexGen LPS-Flex femoral component (Zimmer, Warsaw, IN, USA). Postoperatively the patients were allowed immediate weight-bearing.

For the RSA analysis, 6 tantalum markers were inserted into the polyethylene of the monoblock tibial component in standardized positions, and another 9 markers were spread out in the proximal tibial metaphysis. The initial postoperative RSA examination was performed a mean 3 days (1–5) after the operation. Subsequent examinations were performed at 6 weeks, at 3, 12, and 24 months, and at 5 and 9 years postoperatively. The patients were examined lying supine with the knee placed within a calibration cage (Cage 10, RSA Biomedical, Umeå, Sweden). At the initial postoperative examination the knee was positioned with its anatomical axes parallel to the cardinal axes of the calibration cage.

Analysis of implant migration was performed using UmRSA software (version 6.0; RSA Biomedical, Umeå, Sweden). The relative movements (rotations and translations) of the tibial component were measured in relation to the markers in the proximal tibial metaphysis as the fixed reference segment. The rotations were expressed about the transverse, longitudinal, and sagittal axes. Translations of the tibial tray were measured at 5 standardized positions (Nilsson et al. 1991) at the periphery of the tray. In each implant, the largest negative value for translation along the vertical (y) axis was called maximum subsidence, and the largest positive y-translation was called lift-off. Maximum total point motion (MTPM, Ryd 1986) was defined as the length of the 3-D translation vector of the standardized point at the tibial tray periphery that moved the most. The change in MTPM between 1 and 2 years (Ryd et al. 1995), between 2 and 5 years (Wilson et al. 2012), and between 5 and 9 years was calculated.

The repeatability of the RSA measurements was calculated using double examinations performed postoperatively, and after 1, 2, and 9 years as described by Ranstam et al. (2000). Statistically significant rotations at the 95% significance level were > 0.23° (transverse), > 0.21° (longitudinal), and > 0.21° (sagittal). Corresponding value for translations was > 0.10 mm.

Pre- and postoperative knee alignment was measured as the hip–knee–ankle (HKA) angle, with varus being < 180° and valgus > 180°. Alignment of the tibial component in relation to the tibia was measured as described by Nilsson et al. (1991).

Clinical evaluation was performed using the Knee Society knee score at all follow-ups. At the 9-year follow-up the Knee Injury and Osteoarthritis Outcome Score (KOOS) and Forgotten Joint Score (FJS) (Behrend et al. 2012) were also obtained. In addition, at 9 years the patients were asked to grade their satisfaction of the operation as very satisfied, satisfied, somewhat dissatisfied, or dissatisfied.

The implant–bone interface was analyzed at the 9-year follow-up according to Hayakawa et al. (2014). Type A is defined as new bone formation at the base of the tibial tray and around the pegs, as well as longitudinal trabecular thickening at the distal ends of the pegs. In Type B, there is only longitudinal trabecular thickening at the distal ends of the pegs. Type C is defined as no new bone changes or even existence of radiolucencies.

Not all knees could be analyzed with RSA at all follow-ups (Figure 1). Thus, at 6 weeks and 3 months 1 knee each missed RSA. 1 patient was revised after 3 months, 2 moved abroad, and 1 died of cancer.

### Statistics

The main purpose of the study was to analyze the magnitude and pattern of migration over time. Therefore, we analyzed absolute values of migration parameters for which both negative and positive values were possible. Since the absolute values of rotation, MTPM, and maximum subsidence/lift-off were not normally distributed (according to Shapiro–Wilk test for normality), the data were log transformed to achieve normality. Then mean values and 95% confidence intervals (CI) could be calculated and thereafter re-transformed back to the original scale. This was done in order to facilitate comparison with data on the monoblock TM CR implant published recently (Henricson and Nilsson 2016). In this calculation only data for unilaterally operated knees and the first knee in bilaterally operated patients were used.

For information on individual pattern of migration, data for all knees were displayed in 1 graph (Figure 7).

The clinical results (KOOS, FJS, Knee Society scores) were reported only for the first knee in bilaterally operated persons.

### Ethics, funding, and potential conflicts of interest

The study was approved by the Ethics Committee of Umeå University (entry no. 07-135M). All patients who accepted participation signed an informed consent. The study was not registered at a public registry because the study started in 2007 when registration was still in its early stages. The study was supported by institutional grants from Zimmer-Biomet and Umeå University, Umeå, Sweden. Except for sponsoring the RSA analysis, Zimmer-Biomet had no influence on study planning or implementation. KGN is engaged at Zimmer-Biomet speaker’s bureau, whereas the other authors declare no conflict of interest.

## Results

### Implant migration

**Figure 2.**
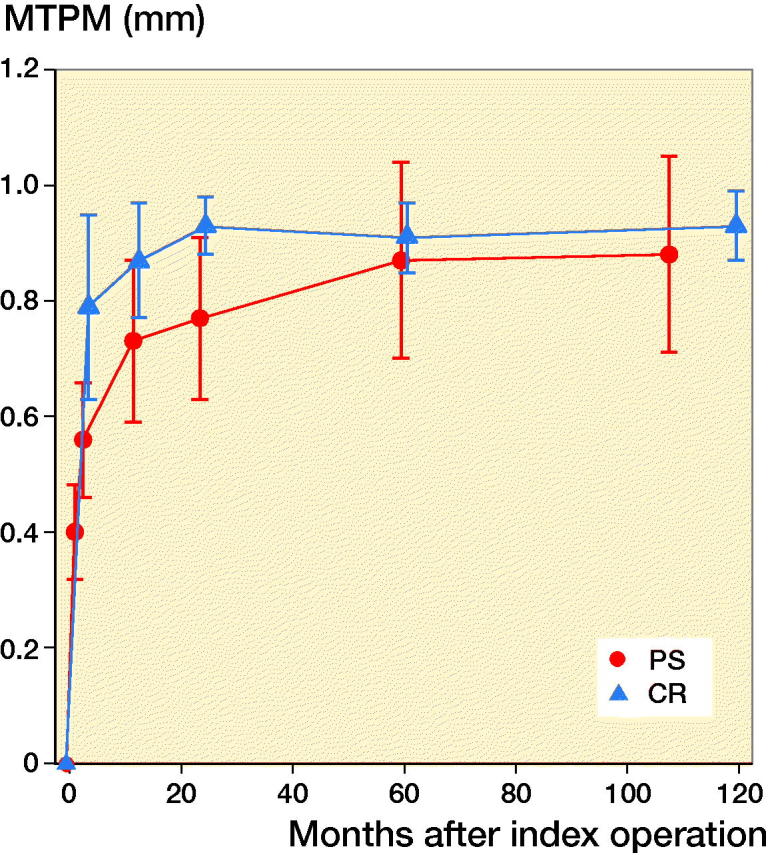
Maximum migration (MTPM) for the NexGen Trabecular Metal posterior stabilized (PS) monoblock tibial component (red). For comparison values for the cruciate retaining (CR) variety of the same implant (blue) (Henricson and Nilsson 2016). Values are mean (95% confidence interval).

**Figure 3.**
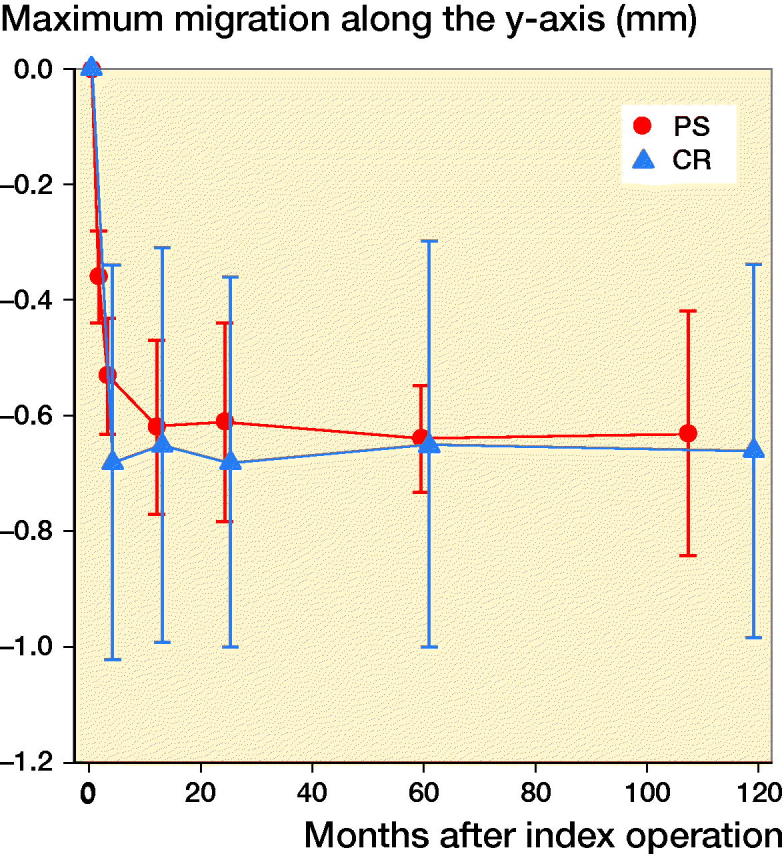
Maximum subsidence (negative y-axis translation) for the NexGen Trabecular Metal posterior stabilized (PS) monoblock tibial component (red). For legend, see Figure 2.

**Figure 4.**
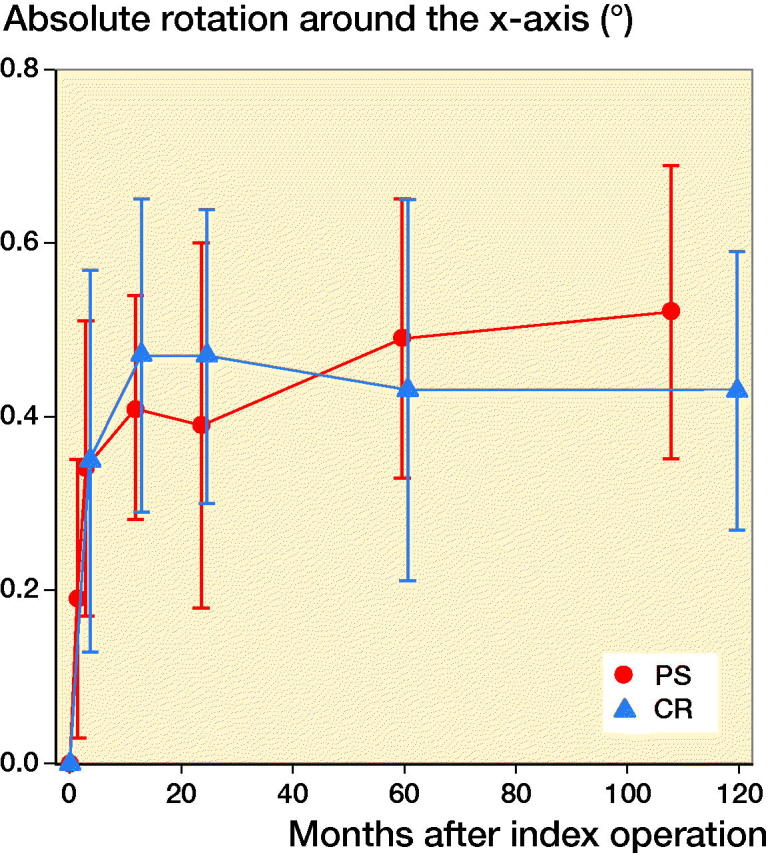
Rotation around the transverse (x-) axis of the knee (absolute values). For legend, see Figure 2. The majority of the TM PS implants rotated into posterior tilt.

**Figure 5.**
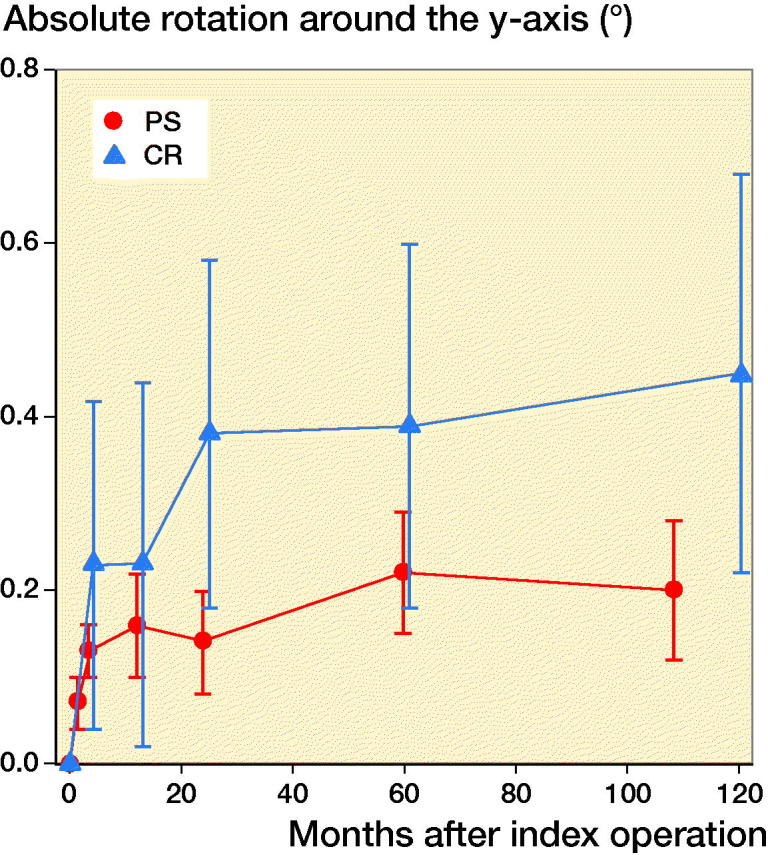
Rotation around the longitudinal (y-) axis of the knee (absolute values). For legend, see Figure 2. The majority of the TM PS implants rotated externally.

**Figure 6.**
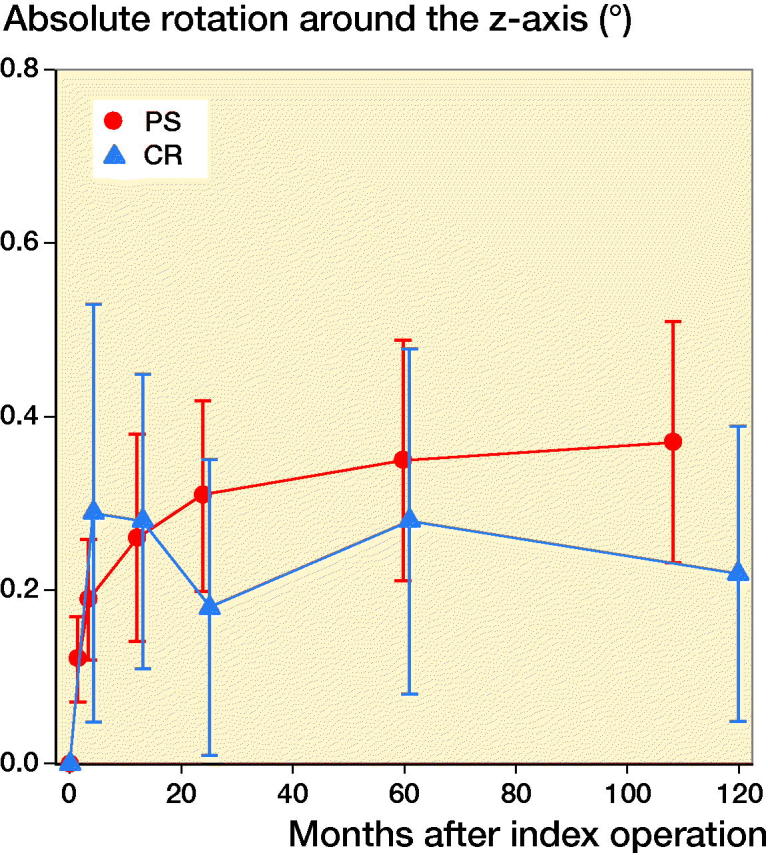
Rotation around the sagittal (z-) axis of the knee (absolute values). For legend, see Figure 2. Rotation into varus or valgus was evenly distributed among the TM PS implants.

**Figure 7.**
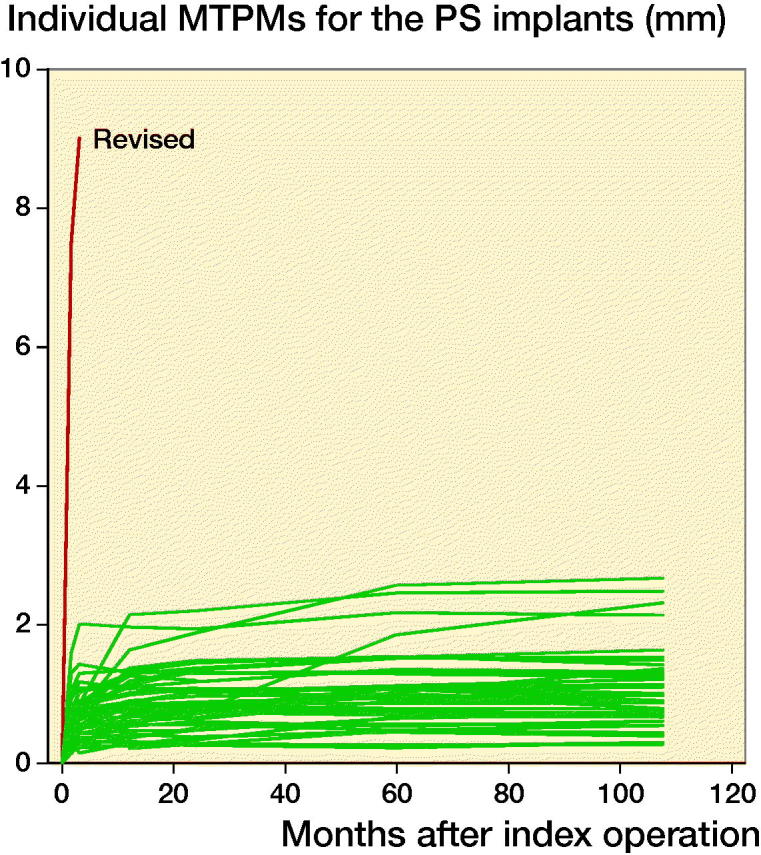
Individual MTPM values for the TM PS implants.

For all migration parameters the pattern of migration was similar, with most of the migration occurring within the initial 3 months and thereafter stabilization over time (Figures 2–6). Compared with the monoblock CR TM implants, the time for stabilization was somewhat longer. The majority of the implants displayed rotation into posterior tilt and external rotation, whereas rotation into varus or valgus was evenly distributed. Rotation around the transverse and sagittal axes was associated with a mean maximum subsidence of the periphery (usually posteriorly) of a mean 0.63 (CI 0.41–0.87) mm (Figure 3). Maximum lift-off of the edge of the tibial tray was small and occurred only occasionally, mostly anteriorly. Between 1 and 2 years one implant displayed MTPM > 0.2 mm, and between 2 and 5 years 4 implants displayed MTPM > 0.3 mm. Between 5 and 9 years all implants except one were stable. Individual MTPM values are shown in Figure 7.

### Radiographic findings

The postoperative alignment of the knee and the tibial component for all implants is given in Table 2. The majority of the knees (n = 37) were aligned 180° ± 3°. In the frontal plane the tibial trays were inserted in slight varus (mean 89° [SD 2.0°]). The mean posterior slope was 6° (SD 2.0°).

**Table 2.** Clinical and radiological data at the 9-year follow-up

Factor	Mean	SD	Median	Range
Knee Osteoarthritis Outcome Score (KOOS) **^a^**				
Symptoms	85	13	86	64–100
Pain	87	15	92	42–100
Function, daily living	86	16	91	35–100
Function, sports and recreational activities	53	31	50	5–100
Quality of life	72	25	81	31–100
Forgotten Joint Score (FJS) **^a^**	65	26	73	23–100
Knee Society Knee Score **^a^**	97	3.9	100	80–100
Knee Society Pain Score ^b^	50	1.3	50	40–50
Knee Society Function Score **^a^**	98	9.2	100	60–100
Extension lag (°)	2	2.2	0	0–15
Flexion (°)	131	10	130	120–145
Knee alignment postop (°) **^c^**	179	3.0	179	172–185
Tibial component				
alignment frontal plane (°) ^d^	89	2.0	89	83–93
posterior slope (°)	6	2.3	6	2–10

**^a^** Maximum 100.

**^b^**50 = no pain

**^c^** Hip–knee–ankle angle, < 180° = varus, > 180° = valgus alignment.

**^d^** < 90° = varus, > 90° = valgus alignment.

At 9 years all but 2 knees displayed Hayakawa Type A implant–bone interface, 1 knee type B, and 1 knee type C.

### Clinical findings

The results for KOOS, FJS, and Knee Society scores at 9 years are listed in Table 2. Of the 36 patients examined, 30 were very satisfied with their knee operation, 4 were satisfied, 1 was somewhat dissatisfied, and 1 was dissatisfied (see also below). Mean knee flexion was 130° and only 1 patient had a slight flexion contracture.

### Complications

1 patient (female, 55 years) with bilateral operations staged 6 months apart had her second (right) operated knee revised 3 months postoperatively. Postoperative knee alignment was 3° varus and tibial component alignment 4° varus. The tibial implant subsided 9 mm medially within the first weeks postoperatively, resulting in a severe varus malalignment. There were no signs of infection. At revision 3 months after the index operation, the tibial implant was firmly fixed to bone and had to be cut out with saw and chisels. Bone underneath and adjacent to the implant showed signs of bone necrosis on microscopic analysis. A stemmed revision tibial component was inserted. The first (left) knee operated on this patient had postoperative HKA angle 180° and tibial component alignment of 1° varus, and functioned very well during the follow-up.

2 patients developed early (within 2 weeks postoperatively) wound complications and peri-prosthetic joint infection, which required early debridement, antibiotics, irrigation, and implant retention (DAIR). Both infections healed. 1 of the patients moved abroad and stated by telephone enquiry 5 and 9 years postoperatively that the knee prosthesis was still in place and functioned well. The other patient scored “very satisfied” at the 9-year follow-up.

## Discussion

This study shows that a TM posterior-stabilized monoblock tibial component together with a high-flex femoral component in patients 60 years or younger displays a benign pattern of migration up to 9 years, i.e., initial migration followed by early stabilization lasting up to 9 years. Mean knee flexion was 130° and the radiological and clinical results were good with 34 of the 36 patients being very satisfied or satisfied with the procedure at the 9-year follow-up.

Most RSA studies have focused on the magnitude of migration during the early postoperative years. Pijls et al. (2012b, 2018), in 2 systematic reviews of mostly cemented implants, found that the magnitude of migration at 6 months and 1 year was associated with rate of late revision. However, several RSA studies in recent years have stressed that the pattern of migration over time is also of importance in evaluating quality of implant fixation (Pijls et al. 2012a, Henricson and Nilsson 2016, Molt et al. 2016, van Hamersveld et al. 2017, 2018). Indeed, Pijls et al. (2018) in their study, albeit advocating only short RSA follow-up to determine long-term performance, stressed that “a particular migration pattern may be normal for one TKA design or fixation, but pathological for another,” indicating a need for longer RSA follow-up.

The migratory pattern for the uncemented monoblock PS TM implant was similar to what has been found for other types of uncemented tibial components (Nilsson et al. 2006, Henricson and Nilsson 2016), with almost all migration occurring during the early postoperative months followed by stabilization. Bellemans (1999) in an RSA study on sheep found that implant stability was compatible with bony ingrowth. The pattern of migration and the radiological findings in the present study thus make it probable that bony ingrowth did occur. Indeed, in the retrieval study of Hanzlik et al. (2013) bony ingrowth was found in all retrieved TM tibial components.

The pattern of migration for the monoblock TM PS implant was similar to its CR counterpart. In registry-based comparisons of cemented PS and CR implants, revision rates have been found to be larger for the PS design (Comfort et al. 2014, Vertullo et al. 2017). This difference in favor of the CR implant has been suggested to be caused by selection bias (Ritter et al. 2014). This statement was, however, refuted by Vertullo et al. (2017), who, by eliminating the selection bias in the analysis, showed cemented PS implants to have larger revision rate than CR implants up to 13 years’ follow-up. This implies that the inferior fixation of the cemented PS implants may be due to the cement mantle not being strong enough to resist the increased load to the implant–bone interface caused by the larger constraint in the PS implants (Catani et al. 2004).

We did not see the potential negative effect of the increased constraint of the PS implant on fixation. The increased forces at the interface in the PS design did not translate into larger migration or lack of stabilization over time. Also, in all but 2 knees there were no radiolucent lines at the implant–bone interface, but rather new bone formation at the base of the tibial tray and around the pegs. This finding may reflect some specific features of how TM interacts with bone. TM has a very high coefficient of friction (Zhang et al. 1999), a porosity, pore size, and modulus of elasticity resembling cancellous bone (Zardiackas et al. 2001, Levine et al. 2006), and ability to stimulate attachment of, and mineralization by, osteoblasts (Sagomonyants et al. 2011), all of which are factors that favors fixation. The resemblance to cancellous bone also makes TM less likely to induce stress shielding (Minoda et al. 2013). The positive results of the monoblock TM implant may, however, not necessarily be valid for modular TM implants, where the tibial tray is a combination of a titanium shell and TM coating, a construct that is less elastic (Zandee van Rilland et al. 2015, Behery et al. 2017).

Male sex, posttraumatic OA, uncemented fixation, and low patient age are all considered risk factors for inferior results after knee arthroplasty (Scott et al. 2015, Watters et al. 2017, Putman et al. 2018). In our study there were equally as many men as women, the mean age was 55 years, all tibial implants were uncemented and PS, and more than half had posttraumatic OA. The low incidence of complications and benign pattern of migration we found indicate that the monoblock TM PS implant may be able to counteract the negative effects of these factors.

There are few articles published on younger persons operated with TKA using KOOS and FJS as patient-reported outcome measures (PROMs). Parratte et al. (2015), in a study comparing total knee arthroplasty with bicondylar knee arthroplasty (medial unicondylar plus patello-femoral implant) in patients with a mean age of 61 and minimum 2-year follow-up, found KOOS results similar to the present study for the total knee arthroplasties. The results for the bicondylar implants were, however, higher. Also, the results for FJS in the total knee arthroplasty group were on a par with our study.

Mean knee flexion in our study was 130° which is much higher than the mean of 110° that was found for the TM CR implants in the previous study (Henricson and Nilsson 2016). Also, extension lag was seldom found. These findings corroborate the results from other studies of better knee flexion in PS designs (Li et al. 2014, Jiang et al. 2016), without the expense of inferior fixation.

2 patients sustained early postoperative infections that were treated with debridement, antibiotics, and implant retention (DAIR); however, due to the monoblock nature of the implant no modular parts could be exchanged. Both infections healed with no signs of recurrent infection during the follow-up. It has been suggested that tantalum may have antimicrobial properties (Schildhauer et al. 2006), but whether this was a contributing factor to the positive outcome in the present study cannot be determined.

There was 1 reoperation of the right knee in a patient who had had the left knee operated 6 months earlier. The patient could not recall a trauma and there were no signs of infection. At revision the implant was firmly fixed to bone, but the bone beneath the tray was necrotic. The reason for this complication is unclear. Similar sized implants were used in both knees. However, in the left knee the knee as well as the tibial component was nearly normally aligned, whereas there was varus alignment of both the knee and tibial component in the right knee. It may as well be that this combined varus malalignment led to medial condyle overload, bone necrosis, and implant subsidence.

The strengths of the study are the long follow-up using RSA on comparatively many knees. Weaknesses are loss of RSA follow-up for various reasons of 4 patients; however, clinical results are lacking for only 1.

In summary, this study shows that the uncemented mono­block TM PS tibial component in patients 60 years or younger yields high knee flexion and displays a pattern of migration up to 9 years indicating a good long-term prognosis as regards fixation and clinical result.

KGN initiated the study, performed the surgery, wrote and edited the manuscript. RW, AH, and SC wrote and edited the manuscript.

*Acta* thanks Stephan Maximilian Röhrl and Sören Toksvig-Larsen for help with peer review of this study.
